# Modeling NO Biotransport in Brain Using a Space-Fractional Reaction-Diffusion Equation

**DOI:** 10.3389/fphys.2021.644149

**Published:** 2021-06-25

**Authors:** Andrew Tamis, Corina S. Drapaca

**Affiliations:** Department of Engineering Science and Mechanics, Pennsylvania State University, University Park, PA, United States

**Keywords:** nitric oxide, anomalous diffusion, fractional calculus, Maxwell viscoelastic model, mechanotransduction

## Abstract

Nitric oxide (NO) is a small gaseous molecule that is involved in some critical biochemical processes in the body such as the regulation of cerebral blood flow and pressure. Infection and inflammatory processes such as those caused by COVID-19 produce a disequilibrium in the NO bioavailability and/or a delay in the interactions of NO with other molecules contributing to the onset and evolution of cardiocerebrovascular diseases. A link between the SARS-CoV-2 virus and NO is introduced. Recent experimental observations of intracellular transport of metabolites in the brain and the NO trapping inside endothelial microparticles (EMPs) suggest the possibility of anomalous diffusion of NO, which may be enhanced by disease processes. A novel space-fractional reaction-diffusion equation to model NO biotransport in the brain is further proposed. The model incorporates the production of NO by synthesis in neurons and by mechanotransduction in the endothelial cells, and the loss of NO due to its reaction with superoxide and interaction with hemoglobin. The anomalous diffusion is modeled using a generalized Fick’s law that involves spatial fractional order derivatives. The predictive ability of the proposed model is investigated through numerical simulations. The implications of the methodology for COVID-19 outlined in the section “Discussion” are purely exploratory.

## Introduction

Nitric oxide (NO) is a small gaseous molecule involved in crucial biochemical processes in the body, especially in the brain. Its relevance to human health can hardly be overestimated. In 1992, Koshland called NO “the molecule of the year” in the editorial of *Science* (Koshland, 1992), and in 1998, Furchgott, Ignarro, and Murad were awarded the Nobel Prize in Physiology or Medicine for its discovery (The Nobel Prize in Physiology or Medicine, 1998). A recent Google search of NO returned more than 35 million results. As the scientific knowledge of the body’s chemophysical processes expands, and technology advances, new discoveries of the role of NO continue to be made.

The major contribution of NO is to the signaling processes of the cardiovascular and nervous systems. In the brain, NO acts as a neuro-glial-vascular messenger regulating cerebral blood flow and the release of neurotransmitters (Moncada et al., 1991; Huang, 1999; Buerk et al., 2003; Ledo et al., 2005; Hall and Garthwaite, 2006; Barbosa et al., 2008; Attwell et al., 2010; Contestabile et al., 2012; Forstermann and Sessa, 2012; Santos et al., 2012; Garry et al., 2015; Helms et al., 2016; Mishra, 2017). Cerebral NO is produced by synthesis reactions within neurons (Forstermann and Sessa, 2012), by shear-induced mechanotransduction at the blood–endothelium interface (Sriram et al., 2016), in the choroid plexus (Garry et al., 2015), and the red blood cells (Helms et al., 2016). Diffusion and some specialized chemical processes [such as NO inactivation by hemoglobin and NO reaction with superoxide ($O_{2}^{-}$)] contribute to the removal of NO from the brain (Helms et al., 2016). There are regions in the brain where the neuronal NO dominates, other regions where the neuronal and endothelial NO are produced in the same amount, and also regions (such as the hippocampus) where only endothelial NO is present (Dinerman et al., 1994). Experiments on knockout mice suggest that if the amount of neuronal (endothelial) NO is lower than expected, its functions could be performed by the endothelial (neuronal) NO (Huang, 1999). Although both the neuronal and endothelial NO are involved in vasomotor mechanisms (Metea and Newman, 2006; Attwell et al., 2010; Contestabile et al., 2012; Mishra, 2017), the endothelial NO is better known as a vasodilator throughout the entire cardiovascular system. In addition, endothelial NO inhibits leukocyte adhesion to the vascular walls and platelet aggregation (Huang, 1999).

**FIGURE 1:**
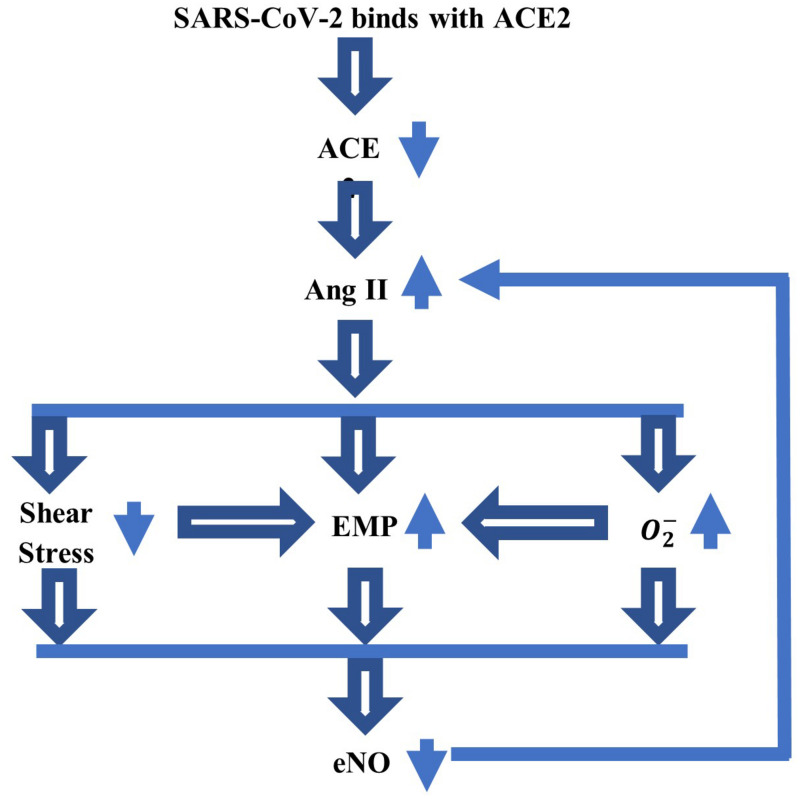
A schematic of the link between SARS-CoV-2 virus and endothelial nitric oxide (NO), which could explain some of the serious cardiovascular and neurological symptoms experienced by a part of patients with COVID-19. The binding between the coronavirus and ACE2 reduces the ACE2 bioavailability, which further increases the amount of Ang II. This increase will lower the shear stress at the blood–endothelium interface and increase the amounts of superoxide ($O_{2}^{-}$) and endothelial microparticles (EMP). The low shear stress and elevated $O_{2}^{-}$ also contribute to the increase in EMP. A decrease in the concentration of endothelial NO (eNO), thus, follows. The feedback mechanism between Ang II and eNO reduces the amount of eNO still further.

Of particular interest this year is the involvement of NO in specific biochemical processes that could lead to the worrisome cardiovascular and neurological symptoms seen in some patients with COVID-19. The SARS-CoV-2 virus binds to the ACE2 protein (Sriram et al., 2020) causing a decrease in ACE2 bioavailability in the body. This decrease in ACE2 produces an increase in angiotensin II (Ang II), a multifunctional peptide hormone, which is followed by a cascade of adverse events, such as a decreased shear stress at the blood–endothelium interface and increased productions of endothelial microparticles (EMPs) and superoxide (Yan et al., 2003; Densmore et al., 2006; Chironi et al., 2009; Burger et al., 2013; Foley and Conway, 2016). Furthermore, the low shear stress and increased amount of superoxide also contribute to the formation of EMP. Not only that there will be less endothelial NO produced by the diminished shear stress but also the endothelial NO bioavailability will be reduced even more by the larger concentration of superoxide that rapidly reacts with the NO to form the peroxynitrite anion. The EMPs are very small fragments of the membrane of endothelial cells that are formed by the blebbing of the plasma membrane and discharged into the extracellular space where they can rapidly move to other sites. They entrap the endothelial NO and, thus, further contribute to the depletion of NO (Chironi et al., 2009; Leopold, 2020). These microparticles can also be formed and contribute to pathological and inflammatory processes of the cardiovascular system (Burger et al., 2013). Also, EMPs can increase the arterial stiffness (Chironi et al., 2009). Last, the feedback mechanism between Ang II and endothelial NO continues to increase the amount of Ang II as the concentration of endothelial NO decreases (Yan et al., 2003). A low amount of endothelial NO will lead to endothelial dysfunction and increased inflammation, thrombosis, and vascular damage. Furthermore, in the regions of the brain where neuronal NO and endothelial NO are both present, the neuronal NO may have to also provide the functions of the endothelial NO when not enough endothelial NO is available, which could cause neurological problems (Huang, 1999). Figure 1 shows a schematic of the abovedescribed link between the SARS-CoV-2 virus and the endothelial NO. The work presented in this article is the first step in building a mathematical model for the mechanism presented in Figure 1.

Mathematical models of NO spatiotemporal dynamics can provide important insights into the role played by NO in the cardiovascular and brain’s functions (Vaughn et al., 1998a, b; Buerk, 2001; Kavdia et al., 2002; Hall and Garthwaite, 2006; Drapaca and Tamis, 2019; Tamis and Drapaca, 2021). In particular, the reaction–diffusion equation of NO proposed in Drapaca and Tamis (2019) and Tamis and Drapaca (2021) models NO biotransport in the brain and, thus, incorporates terms for the production and decay of both endothelial and neuronal NO. The shear-induced production of endothelial NO is assumed to be proportional to the concentration of endothelial NO with the production rate given by the viscous dissipation at the blood–endothelium interface. The viscous dissipation is calculated from mechanical models of blood flow and vascular wall deformability [for instance, in Drapaca and Tamis (2019), the blood flow is Poiseuille, and the vascular wall is rigid, while in Tamis and Drapaca (2021), the viscoelastic wall is subjected to pulsatile blood flow]. The production of neuronal NO is assumed to be constant, and only the NO decay due to the hemoglobin is considered.

In this article, a novel space-fractional reaction–diffusion equation for the NO biotransport in the brain is proposed. The model assumes anomalous diffusion of NO, which could be enhanced by the presence of pathology. The trapping of NO by EMP mentioned above supports the assumption of anomalous diffusion (subdiffusion) of NO at least in pathological conditions (in normal conditions, the anomalous diffusion of endogenous intracellular metabolites in the brain observed *in vivo* using medical imaging techniques (Marchadour et al., 2012) could suggest a similar diffusive behavior for the NO, which is known to modulate the transport of some of these metabolites (Raju et al., 2015)). In this model, the presence of EMP will be represented by the anomalous diffusion of NO. Anomalous diffusion in various engineering and biological systems has been successfully modeled using fractional calculus (Metzler and Klafter, 2004; Kim and Kavvas, 2006; Klages et al., 2008; Magin et al., 2008, 2013; Tarasov, 2010; Zhou et al., 2010; Dumitru et al., 2012; West, 2016, 2017; Liang et al., 2017; Drapaca, 2021), and thus, spatial derivatives of a fractional order of 0 < ε < 1 will be used in this mathematical model. The link between anomalous diffusion and fractional-order derivatives is based on the interpretation of anomalous diffusion as a Lévy flight process: the jump length probability density function (PDF) of a Lévy flight is a power law of exponent −(ε + 1) (the classic Gaussian distribution is recovered for ε = 1) and is solution to a space-fractional differential equation [see for instance, theorem 6.14 in Dumitru et al. (2012)]. In this article, left- and right-sided fractional Riemann–Liouville derivatives of order ε model bidirectional diffusion. Two probabilities, *p*, *q* ≥ 0 with *p* + *q* = 1, for forward and, respectively, backward diffusional directions are introduced to model NO distribution skewness (Kim and Kavvas, 2006). The production of neuronal NO is assumed to be time dependent so that it matches the kinetics of Ca^2+^-calmodulin binding (Hall and Garthwaite, 2006), while the shear-induced production of endothelial NO is calculated as in Tamis and Drapaca (2021). Thus, the vascular wall is assumed to be a homogeneous, Maxwell linear viscoelastic material subjected to the heart pulsations (Hodis and Zamir, 2008; Tamis and Drapaca, 2021). Last, the model incorporates the NO decay due to hemoglobin and NO reaction with superoxide. The proposed equation is solved numerically using the numerical scheme proposed in Sousa (2009). Numerical simulations show the effects of anomalous diffusion, increased vascular stiffness, and increased amount of superoxide on the spatiotemporal distribution of NO concentration. The results suggest the existence of a narrow range of values of ε near 0.85 where a maximum value of the NO concentration at the endothelium is reached that decreases with an increase in vascular stiffness and/or a rise in the amount of superoxide. Also, the results show an increase in the distribution skewness of the NO at the endothelium for ε in the same range. Thus, the model reveals the existence of a characteristic jump length distribution where the spatial correlation (nonlocality) of NO particles happens due to a critical (possibly elevated) quantity of EMP present such that, when combined with a preferred forward diffusional direction, a significant decrease in the NO concentration at the vascular wall occurs in the presence of an increased vascular stiffness and amount of superoxide. Given the functional interplay and feedback mechanism between Ang II and endothelial NO (Chironi et al., 2009; Leopold, 2020), any untreated disease that produces an increase in the amount of Ang II above a healthy threshold (such as possibly COVID-19, diabetes, and cardiovascular and neurodegenerative diseases) will lead, in time, to an increase in both vascular rigidity and concentration of superoxide, whose combined contribution to the decrease in the NO concentration will ultimately cause endotheliopathy. The proposed model predicts this behavior in the presence of the anomalous diffusion of NO with ε = 0.85. Also, according to Hall and Garthwaite (2006), physiological concentrations of NO are in the low *nM* (or 10^−6^ *mol*/*m*^3^) range. The model with classic diffusion (ε = 1) predicts maximum NO concentrations at the vascular wall in the low *pM* range, while the analogous values for ε < 1 belong to the physiological range. Last, given how small these concentrations are even in a healthy state, it may be difficult to measure/estimate them in human subjects. For ε = 0.85, the model found a maximum NO concentration at the wall corresponding to a vascular wall stiffness and amount of superoxide within normal ranges. This information could be used not only for noninvasively imaging the NO *in vivo* but also for controlled drug delivery (see section “Discussion”).

The structure of the article is as follows. The mathematical model is presented in the section “Mathematical Model,” while the corresponding numerical scheme is given in the section “Numerical Scheme.” Numerical simulations are shown in the section “Results,” which is followed by a section “Discussion.” The article ends with a section “Conclusion and Future Work.”

## Mathematical Model

**FIGURE 2:**
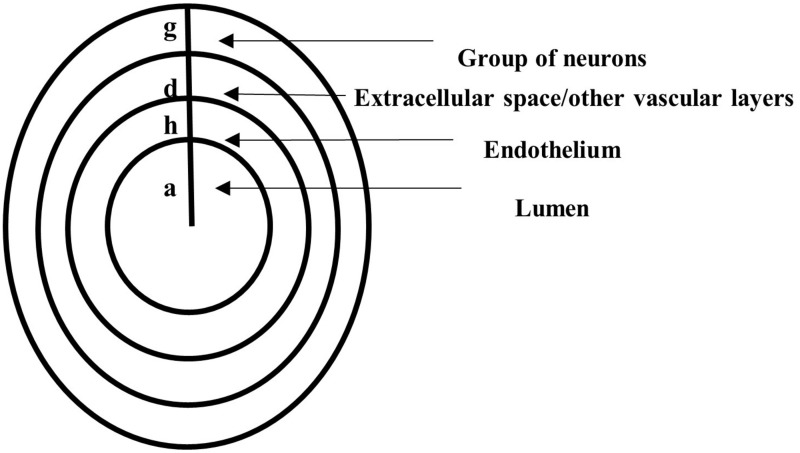
A vertical cross-section through concentric horizontal axial symmetric circular cylinders representing the lumen of radius *a*, the endothelium of thickness *h*, a region of thickness *d* filled with other vascular layers and extracellular space, and a layer of thickness *g* occupied by neurons.

The geometric domain is made of concentric horizontal axial symmetric circular cylinders (Figure 2). The lumen occupies a cylinder of radius *a*. The endothelium is a layer of thickness *h*, and other vascular structures and the extracellular space fill a region of thickness *d*. Last, a group of neurons occupies a region of thickness *g*. The geometric assumptions imply spatial variations of NO in the radial direction only. Cylindrical coordinates are used with *x*, the axial coordinate along the horizontal direction, and *r* ∈ [0, *a* + *h* + *d* + *g*], the radial coordinate along the radial direction (due to axial symmetry, there is no dependency on the angular coordinate, and thus, this coordinate is neglected).

As in Drapaca and Tamis (2019) and Tamis and Drapaca (2021), it is assumed that the radial diffusion of NO happens in the entire domain *r* ∈ [0, *a* + *h* + *d* + *g*]. NO is produced in the region *r* ∈ [*a*, *a* + *h*]∪[*a* + *h* + *d*, *a* + *h* + *d* + *g*], and NO decay due to nondiffusional biochemical processes happens in the region *r* ∈ [0, *a*]∪[*a* + *h*, *a* + *h* + *d* + *g*]. The mathematical model expresses the balance law of mass for the concentration of NO, *C*_*NO*_:

(1) $$\begin{array}{c} \frac{\partial C_{NO}}{\partial t}=-\frac{1}{r}\frac{\partial }{\partial r}\left(rF\left(r,t\right)\right)+\left[v_{1}\left(1-e^{-k_{1}t}\right)e^{k_{2}t}\right]\\ \;H\left(r-\left(a+h+d\right)\right)-\frac{v_{max}C_{NO}}{K_{max}+C_{NO}}H\left(r-\left(a+h\right)\right)\\ \;+\frac{\sigma_{rx}}{\tau_{W}}\frac{\partial \varepsilon_{rx}}{\partial t}\left[H\left(r-a\right)-H\left(r-\left(a+h\right)\right)\right]\\ \;C_{NO}-\lambda \left[1-H\left(r-a\right)\right]C_{NO}\\ \;-\tilde{\lambda }\left[H\left(r-\left(a+h\right)\right)-H\left(r-\left(a+h+d\right)\right)\right]C_{NO} \end{array}$$

Above, *H* denotes the Heaviside function.

Equation (1) is solved numerically (see later) with zero initial conditions and zero Dirichlet boundary conditions:

(2) $$C_{NO}\left(r,0\right)=0,C_{NO}\left(0,t\right)=C_{NO}\left(a+h+d+g,t\right)=0$$

The right-hand side terms of equation (1) are as follows. The diffusion flux *F*(*r*, *t*) is given by the generalized Fick’s law describing anomalous (or nonlocal) diffusion [see for instance, Kim and Kavvas (2006), for a derivation of this law]:

(3) $$F\left(r,t\right)=-D_{NO}\left[p\partial_{L+}^{\varepsilon }C_{NO}\left(r,t\right)-q\partial_{R-}^{\varepsilon }C_{NO}\left(r,t\right)\right]$$

In formula (3), *D*_*NO*_ is a generalized diffusion coefficient, 0 < ε ≤ 1 is a parameter that measures the amount of long-range spatial correlation of NO particles, and *p*, *q* ≥ 0 are probabilities of moving in the forward and, respectively, in the backward diffusional direction. Probabilities *p*, *q*satisfy the constraint *p* + *q* = 1. By definition, the left- and right-sided Riemann–Liouville fractional derivatives of order ε with 0 < ε ≤ 1 are [see for example, Metzler and Klafter (2004) and references within]:

(4) $$\partial_{L+}^{\varepsilon }C_{NO}\left(r,t\right)=\frac{1}{\Gamma \left(1-\varepsilon \right)}\frac{\partial }{\partial r}\int_{L}^{r}\frac{C_{NO}\left(\tilde{r},t\right)}{{\left(r-\tilde{r}\right)}^{\varepsilon }}d\tilde{r}$$

(5) $$\partial_{R-}^{\varepsilon }C_{NO}\left(r,t\right)=\frac{1}{\Gamma \left(1-\varepsilon \right)}\frac{\partial }{\partial r}\int_{r}^{R}{\frac{C_{NO}\left(\tilde{r},t\right)}{\left(\tilde{r}-r\right)}}^{\varepsilon }d\tilde{r}$$

where, $\Gamma \left(s\right)=\int_{0}^{\infty }t^{s-1}e^{-t}dt$ is the gamma function. The following notations are used throughout the article: *L* = 0, *R* = *a* + *h* + *d* + *g*. For ε = 1, the Riemann–Liouville fractional-order derivatives (4) and (5) become the first-order derivative, and in this case, *p* = *q* = 1/2, and formula (3) reduces to the classic Fick’s law.

The second term of the right-hand side of equation (1) represents the time-dependent production by synthesis of the neuronal NO for *r* ∈ [*a* + *h* + *d*, *a* + *h* + *d* + *g*], which was proposed in Hall and Garthwaite (2006). Parameter *v_1* is the maximum production rate, and parameters *k_1* and *k_2* are chosen to match the kinetics of Ca^2+^-calmodulin binding.

The third, fifth, and sixth terms of the right-hand side of equation (1) represent the NO decay due to various inactivation processes. The inactivation of NO achieved through processes independent of hemoglobin, and superoxide is characterized by *v*_*max*_, the maximum rate at saturating concentration in the region *r* ∈ [*a* + *h*, *a* + *h* + *d* + *g*], and *K*_*max*_, the NO concentration at which the reaction rate is $\frac{v_{max}}{2}$ in the same region. The decay of NO due to blood’s hemoglobin happens at constant rate λ in the region *r* ∈ [0, *a*]. The removal of NO due to its reaction with the superoxide is assumed to happen at constant rate $\tilde{\lambda }$ in the region *r* ∈ [*a* + *h*, *a* + *h* + *d*]. The region where the reaction between NO and superoxide takes place was chosen based on experimental observations suggesting that there is very little amount of superoxide inside the cells (less than 100 pM), and thus, this reaction is unlikely to happen intracellularly (Ganesana et al., 2012).

Last, the fourth term of the right-hand side of equation (1) models the production via shear-induced mechanotransduction of the endothelial NO for *r* ∈ [*a*, *a* + *h*], which was proposed in Tamis and Drapaca (2021). The production rate is assumed to be proportional to the viscous dissipation at the blood–endothelium interface $\frac{\sigma_{rx}}{\tau_{W}}\frac{\partial \varepsilon_{rx}}{\partial \text{t}}$, where σ_*rx*_ and ε_*rx*_ are the shear stress and, respectively, the infinitesimal strain of the endothelium, and τ_*W*_ = σ_*rx*_(*a*, *t*). As in Tamis and Drapaca (2021), the endothelium is assumed to be a homogeneous, Maxwell linear viscoelastic material exposed to the pulsatile blood flow at *r* = *a* and tethered at *r* = *a* + *h*. Thus, the constitutive equation of the viscoelastic endothelium is:

$$\frac{\partial \sigma_{rx}}{\partial t}=E\left(\frac{\partial \varepsilon_{rx}}{\partial t}-\frac{\sigma_{rx}}{\mu }\right)$$

where, *E* is the modulus of elasticity, and μ is the viscosity of the endothelium. The expressions of σ_*rx*_ and ε_*rx*_ are derived from the analytic solution to the following boundary value problem (Hodis and Zamir, 2008):

$$\rho \frac{\partial^{3}\xi }{\partial t^{3}}-E\frac{\partial^{3}\xi }{\partial t\partial r^{2}}+\frac{\rho \omega }{\gamma }\frac{\partial^{2}\xi }{\partial t^{2}}=0$$

$$\xi \left(a,t\right)=\xi_{0}e^{i\omega t},\xi \left(a+h,t\right)=0$$

Where, γ = $\frac{\omega \mu }{E}$, ξ(*r*, *t*) is the axial displacement of the endothelium, ρ is its mass density, ω, and ξ_0_ are the angular frequency and, respectively, amplitude of the applied oscillations. Thus (Tamis and Drapaca, 2021):

$$\frac{\sigma_{rx}}{\tau_{W}}\frac{\partial \varepsilon_{rx}}{\partial t}=\frac{-i\xi_{0}\omega \left(\alpha^{2}+\beta^{2}\right)\left[e^{2\alpha \left(h-r+a\right)}+e^{-2\alpha \left(h-r+a\right)}+2\right]}{\left(\alpha -i\beta \right)e^{2\alpha h}-\left(\alpha +i\beta \right)e^{-2\alpha h}+2i\beta }$$

(6) $$e^{i\left[\omega t-2\beta \left(r-a\right)\right]}$$

with:

$$\alpha^{2}=\frac{\rho \omega^{2}}{2E}\left[\sqrt{1+\frac{1}{\gamma^{2}}}-1\right],\beta^{2}=\frac{\rho \omega^{2}}{2E}\left[\sqrt{1+\frac{1}{\gamma^{2}}}+1\right]$$

Replacing formula (3) in the first term of the right-hand side of equation (1), and using the product rule for first-order derivatives and the fact that $\partial /\partial r\left(\partial_{L+}^{\varepsilon }C_{NO}\right)=\partial_{L+}^{\varepsilon +1}C_{NO}$, $\partial /\partial r\left(\partial_{R-}^{\varepsilon }C_{NO}\right)=-\partial_{R-}^{\varepsilon +1}C_{NO}$ give:

(7) $$\begin{array}{c} \frac{1}{r}\frac{\partial }{\partial r}\left(rF\left(r,t\right)\right)=-D_{NO}[\left(p\partial_{L+}^{\varepsilon +1}C_{NO}+q\partial_{R-}^{\varepsilon +1}C_{NO}\right)\\ \;+\frac{1}{r}\left(p\partial_{L+}^{\varepsilon }C_{NO}-q\partial_{R-}^{\varepsilon }C_{NO}\right)] \end{array}$$

Equation (1) combined with expressions (6) (its real part) and (7), together with the initial and boundary conditions (2), is solved numerically using MATLAB (Matlab, 2020).

## Numerical Scheme

Numerical discretization schemes of the Riemann–Liouville fractional derivatives in formula (7) are given next. There exist numerous numerical schemes for fractional-order operators and a comprehensive review of them can be found in Cai and Li (2020). The classic finite difference schemes used, for instance, in Sousa (2009) are used here. Let *L* = *r*_0_ < *r*_1_ < … < *r*_*N*−1_ < *r*_*N*_ = *R* be an equally spaced discretization of the interval [*L*, *R*] of constant step size Δ*r* = (*R* − *L*)/*N*. The spatial fractional derivatives of order 0 < ε ≤ 1 at nodes *r*_*k*_, *k* = 1,…, *N* − 1 are approximated using the right and left classic Grünwald–Letnikov formulas:

(8) $$\partial_{L+}^{\varepsilon }C_{NO}\left(r_{k},t\right)=\frac{1}{{\left(\Delta r\right)}^{\varepsilon }}\sum_{j=0}^{k}g_{j}^{\varepsilon }C_{NO}\left(r_{k-j},t\right)$$

(9) $$\partial_{R-}^{\varepsilon }C_{NO}\left(r_{k},t\right)=\frac{1}{{\left(\Delta r\right)}^{\varepsilon }}\sum_{j=0}^{N-k}g_{j}^{\varepsilon }C_{NO}\left(r_{k+j},t\right)$$

The right- and left-shifted Grünwald–Letnikov formulas are used to approximate the spatial fractional derivatives of order 1 < ε + 1 ≤ 2 at nodes *r*_*k*_, *k* = 1,…, *N* − 1 since these give a stable numerical scheme:

(10) $$\partial_{L+}^{\varepsilon +1}C_{NO}\left(r_{k},t\right)=\frac{1}{{\left(\Delta r\right)}^{\varepsilon +1}}\sum_{j=0}^{k+1}g_{j}^{\varepsilon +1}C_{NO}\left(r_{k-j+1},t\right)$$

(11) $$\partial_{R-}^{\varepsilon +1}C_{NO}\left(r_{k},t\right)=\frac{1}{{\left(\Delta r\right)}^{\varepsilon +1}}\sum_{j=0}^{N-k+1}g_{j}^{\varepsilon +1}C_{NO}\left(r_{k+j-1},t\right)$$

In formulas (8)–(11), the following notations are used:

$$g_{j}^{\varepsilon }=\frac{{\left(-1\right)}^{j}\Gamma \left(\varepsilon +1\right)}{\Gamma \left(j+1\right)\Gamma \left(\varepsilon -j+1\right)},g_{j}^{\varepsilon +1}=\frac{{\left(-1\right)}^{j}\Gamma \left(\varepsilon +2\right)}{\Gamma \left(j+1\right)\Gamma \left(\varepsilon -j+2\right)}$$

The Grünwald–Letnikov approximations have an error of order Δ*r*^2^. Formulas (8)–(11) highlight the nonlocal effect of the fractional-order derivatives: the numerical approximations of these derivatives at the node *r_k_* do not involve only the neighborhood nodes *r*_*k*–1_ and *r*_*k*+1_ but also farther away nodes.

Formulas (8)–(11) can be rewritten in matrix form by changing the indexes of the terms in the sums of these formulas. Thus, the numerical discretization of formula (7) at nodes *r*_*k*_, *k* = 1,…, *N* − 1 is:

(12) $$\begin{array}{c} \frac{1}{r}\frac{\partial }{\partial r}\left(r_{k}F\left(r_{k},t\right)\right)=-D_{NO}[(p\frac{1}{\Delta r^{\varepsilon +1}}\sum_{j=1}^{N-1}B_{jk}C_{NO}\left(r_{j},t\right)\\ \;+q\frac{1}{\Delta r^{\varepsilon +1}}\sum_{j=1}^{N-1}B_{kj}C_{NO}\left(r_{j},t\right))\\ \;+\frac{1}{r}(p\frac{1}{\Delta r^{\varepsilon }}\sum_{j=1}^{N-1}A_{jk}C_{NO}\left(r_{j},t\right)\\ \;-q\frac{1}{\Delta r^{\varepsilon }}\sum_{j=1}^{N-1}A_{kj}C_{NO}\left(r_{j},t\right))] \end{array}$$

Where:

$$A_{jk}=\left\{\begin{array}{cc} g_{k-j}^{\varepsilon }, & j\leq k\\ 0, & otherwise \end{array}\right\},B_{jk}=\left\{\begin{array}{cc} g_{k-j+1}^{\varepsilon +1}, & j\leq k+1\\ 0, & otherwise \end{array}\right\}$$

For ε = 1, the left-hand side of formula (12) becomes the Laplace operator, which admits only three numerical approximations: a forward finite difference scheme for *p* = 1, *q* = 0, a backward finite difference scheme for *p* = 0, *q* = 1, and a central finite difference scheme for *p* = *q* = 1/2.

By writing equation (1) at the nodes *r*_*k*_, *k* = 1,…, *N* − 1 and using scheme (12), a system of ordinary differential equations is obtained that is solved numerically using MATLAB’s built-in function ode15s. The system is solved using the non-negative option of ode15s since negative values of *C*_*NO*_ are unphysical.

## Results

The parameters used in the numerical simulations are given in Table 1. Numerical simulations show the effects of parameters $\varepsilon ,p,q,\tilde{\lambda },E$, and γ on the spatiotemporal distribution of NO concentration. According to Hodis and Zamir (2008), a healthy vascular wall is characterized by γ = 0.1÷0.25 and *E* = 6 × 10^3^ *N*/*m*. The wall loses its viscoelasticity and becomes more rigid as both mechanical parameters, and *E*, increase. The endothelium is assumed to be slightly more elastic, and thus, the values γ = 0.75 and *E* = 6 × 10^3^ *N*/*m* are used in the numerical simulations. The values for a rigid vascular wall proposed in Hodis and Zamir (2008) are *E* = 6 × 10^4^ *N*/*m* and γ = 1.25, and they are used to investigate how the NO concentration at endothelium changes with increased vascular stiffness in the presence of anomalous diffusion. The rate $\tilde{\lambda }$ is calculated by the following formula:

$$\tilde{\lambda }=k_{O_{2}^{-}}C_{O_{2}^{-}}$$

**TABLE 1:** List of parameters with corresponding values and units.

Considerations	Parameters	Values and units [references]
Geometry	*a* *h* *d* *g*	25 × 10^−6^m (Kavdia et al., 2002) 0.5 × 10^−6^m (Kavdia et al., 2002) 4 × 10^−6^m (Tamis and Drapaca, 2021) 5 × 10^−6^m (Hall and Garthwaite, 2006)
NO diffusion, synthesis, and inactivation	D_*NO*_ ε *p*, *q* *v*_1_ *k*_1_ *k*_2_ *v*_*max*_ $K_{max}\lambda \tilde{\lambda }$	3.3 × 10^−9^m^ε+1^/s (Kavdia et al., 2002; Hall and Garthwaite, 2006) 0.75 ÷ 1 0 ÷ 1, 1 ÷ 0 1.6 × 10^−3^mol/(m^3^ × s) (Hall and Garthwaite, 2006) 2*s*^−1^ (Hall and Garthwaite, 2006) 1.5 s^−1^ (Hall and Garthwaite, 2006) 2 × 10^−3^mol/(m^3^ × *s*) (Hall and Garthwaite, 2006) 10^−5^mol/m^3^(Hall and Garthwaite, 2006) 2.3 × 10^2^ s^−1^ (Vaughn et al., 1998a) 6.5325 × 10^3^ s^−1^ (13.065 × 10^3^*s*^−1^)
Maxwell viscoelastic endothelium	ρ *E*. ω ξ_0_ γ	1kg/*m*^3^. (Tamis and Drapaca, 2021) 6 × 10^3^N/m(6 × 10^4^N/m) (Hodis and Zamir, 2008) 1 Hz (Hodis and Zamir, 2008) 10^−3^m (Tamis and Drapaca, 2021) 0.75 (1.25) (Hodis and Zamir, 2008)

where, the reaction rate is $k_{O_{2}^{-}}=6.7\times 10^{9}M^{-1}s^{-1}$ (Akaike and Maeda, 2000), and the concentration of superoxide in the extracellular space is $C_{O_{2}^{-}}=1.95\times 10^{-6}M$ in normal conditions and about $C_{O_{2}^{-}}=3.9\times 10^{-6}M$ in ischemic conditions (Ganesana et al., 2012). However, since the superoxide is scavenged by the high concentrations of superoxide dismutase (SOD) (Hall and Garthwaite, 2006), only half of the concentrations of superoxide mentioned above are assumed to react with NO, and the corresponding rates $\tilde{\lambda }$ are, thus, calculated (see Table 1). The value $\tilde{\lambda }=13.065\times 10^{3}s^{-1}$ is used to investigate how an increased amount of superoxide influences the NO concentration at the endothelium in the presence of anomalous diffusion. The numerical simulations use a space step size Δ*r* = 1.15×10^−7^ (300 nodes) and a time step size Δ*t* = 0.0251 (400 time points).

**FIGURE 3:**
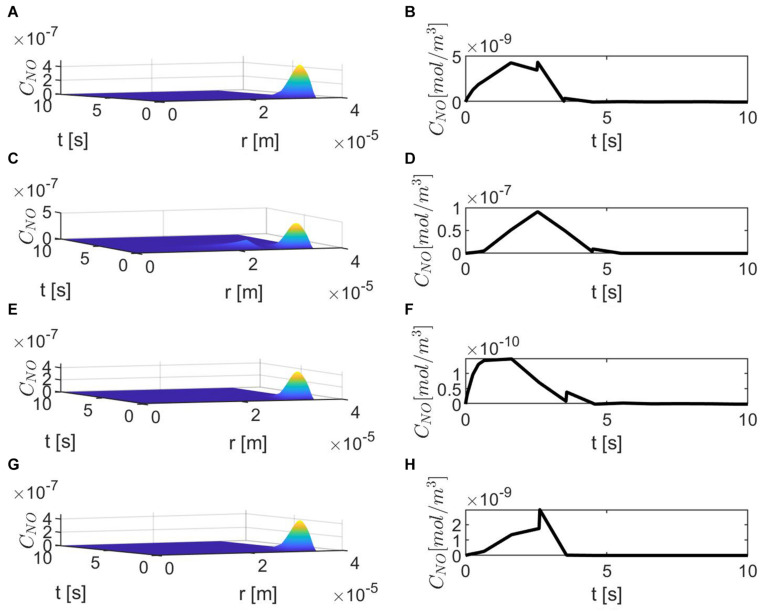
Spatiotemporal variations of *C*_*NO*_ **(A,C,E,G)** and its corresponding temporal profiles at *r* = *a* + *h*, the outer boundary of the endothelium, **(B,D,F,H)** for ε = 1, *p* = *q* = 0.5, **(A,B)** $E=6\times 10^{3}N/m,\gamma =0.75,\tilde{\lambda }=6.5325\times 10^{3}s^{-1}$, **(C,D)** $E=6\times 10^{4}N/m,\gamma =1.25,\tilde{\lambda }=6.5325\times 10^{3}s^{-1}$, **(E,F)** $E=6\times 10^{3}N/m,\gamma =0.75,\tilde{\lambda }=13.065\times 10^{3}s^{-1},$ and **(G,H)** $E=6\times 10^{4}N/m,\gamma =1.25,\tilde{\lambda }=13.065\times 10^{3}s^{-1}.$

Figure 3 shows the results for the case ε = 1, *p* = *q* = 0.5 and various values of *E*, γ, and $\tilde{\lambda }$. The production of neuronal NO is bigger than the one at the endothelium, and this can be seen in Figures 3A,C,E,G. When the concentration of superoxide is increased (larger $\tilde{\lambda }$), the model predicts a bigger decrease in the NO concentration at the endothelium (Figures 3F,H) than when the amount of superoxide is normal (Figure 3B). However, when the stiffness of the vascular wall increases, the production of the endothelial NO increases, and the model predicts an increase in the concentration of NO at the endothelium (Figures 3C,D). Thus, without accounting for a mechanism for increasing vascular stiffness (such as spatial correlation of NO particles due to the presence of EMP), the model cannot predict the decrease in NO concentration due to elevated vascular stiffness mentioned in the literature (Chironi et al., 2009). Also, in this case, the maximum NO concentrations at the vascular wall are in the low *pM* range, which are smaller than physiological values that, according to Hall and Garthwaite (2006), are in the low *nM* range.

**FIGURE 4:**
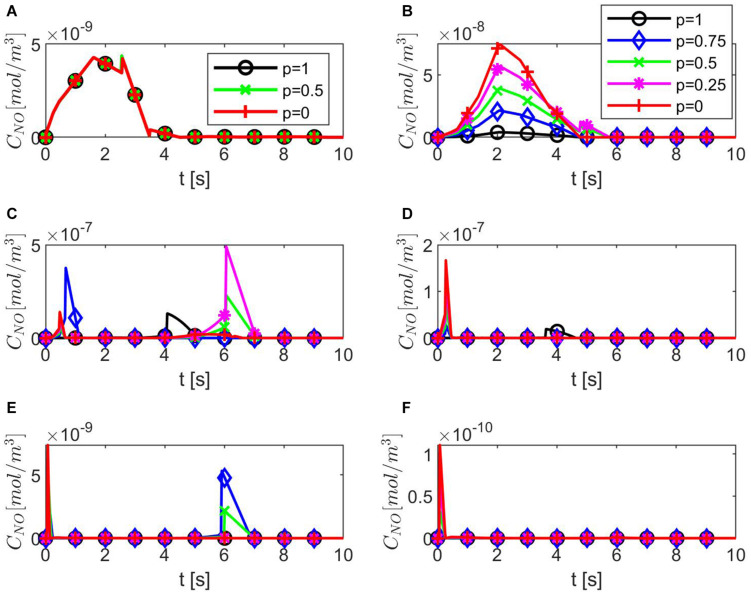
Temporal profiles of *C*_*NO*_ at *r* = *a* + *h*, the outer boundary of the endothelium, for **(A)** ε = 1, **(B)** ε = 0.95, **(C)** ε = 0.85, **(D)** ε = 0.75, **(E)** ε = 0.5, and **(F)** ε = 0.25 and various values for *p* and *q* = 1 − *p* (**B–F** have the same legend shown in **B**). Here $E=6\times 10^{3}N/m,\gamma =0.75,\tilde{\lambda }=6.5325\times 10^{3}s^{-1}.$

**FIGURE 5:**
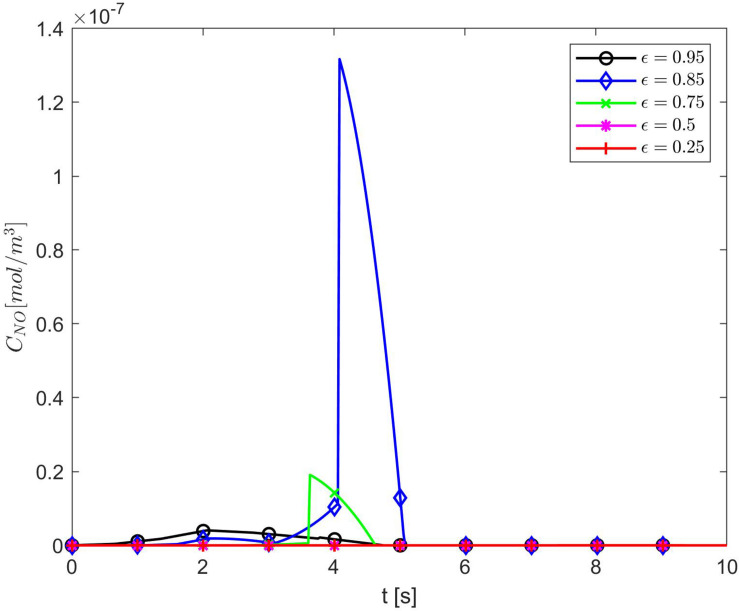
Temporal profiles of *C*_*NO*_ at *r* = *a* + *h*, the outer boundary of the endothelium, for *p* = 1 (*q* = 0) and various values of ε. Here $E=6\times 10^{3}N/m,\gamma =0.75,\tilde{\lambda }=6.5325\times 10^{3}s^{-1}.$

Figure 4 shows temporal profiles of the NO concentration for various values of ε, *p*, *q* when the values of *E*, γ, and $\tilde{\lambda }$ are kept fixed at their values in normal conditions. As expected, in the case ε = 1, the forward (*p* = 1, *q* = 0), backward (*p* = 0, *q* = 1), and center (*p* = *q* = 0.5) finite differences give the same result (Figure 4A). As ε decreases, an increase in the distribution skewness of the NO at the endothelium is observed (Figures 4B–F). For values of ε away from 0.85, the NO concentration at the endothelium increases as *p* decreases from 1 to 0 (or *q* increases from 0 to 1) within the first 4 s (Figures 4B,D–F). For ε close to 0.85, a maximum skewness distribution is observed (Figure 4C). Last, as ε decreases from 1 to 0, the NO concentration at endothelium increases until ε is close to 0.85 and then decreases (Figures 4A–F, 5). Figure 5 also shows that as ε decreases to 0.85, the peak of the NO concentration becomes slightly delayed in time for fixed *p* and *q*. These results suggest that for ε near 0.85, there is enough NO present in the endothelium and an adequate amount of EMP available such that changes in the NO concentration due to a stiffer vascular wall and/or larger quantity of superoxide can be observed.

**FIGURE 6:**
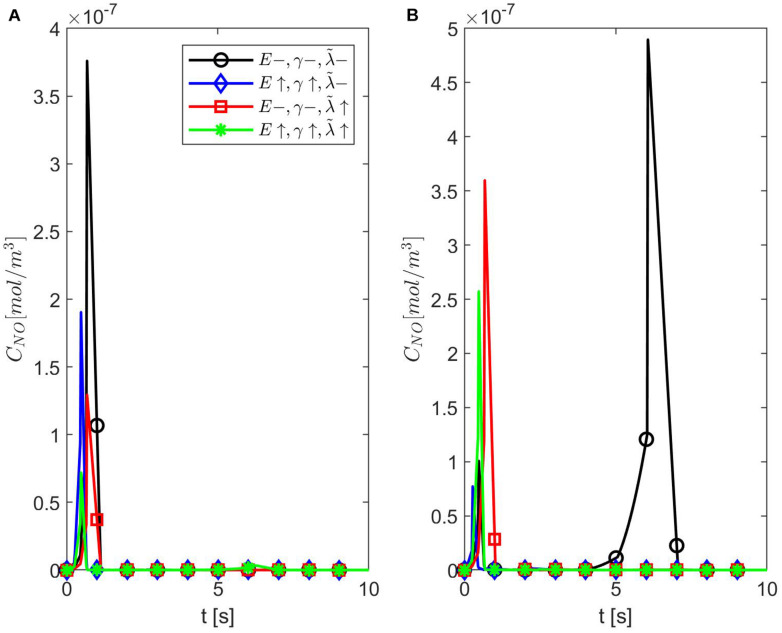
Temporal profiles of *C*_*NO*_ at *r* = *a* + *h*, the outer boundary of the endothelium, for ε = 0.85, **(A)** *p* = 0.75 (*q* = 0.25), and **(B)** *p* = 0.25 (*q* = 0.75) (**A,B** have the same legend shown in **A**). Here $E=6\times 10^{3}N/m,\gamma =0.75,\tilde{\lambda }=6.5325\times 10^{3}s^{-1}$ (black circle symbol), $E=6\times 10^{4}N/m,\gamma =1.25,\tilde{\lambda }=6.5325\times 10^{3}s^{-1}$ (blue diamond symbol), $E=6\times 10^{3}N/m,\gamma =0.75,\tilde{\lambda }=13.065\times 10^{3}s^{-1}$ (red square symbol), and $E=6\times 10^{4}N/m,\gamma =1.25,\tilde{\lambda }=13.0655\times 10^{3}s^{-1}$ (green star symbol).

**FIGURE 7:**
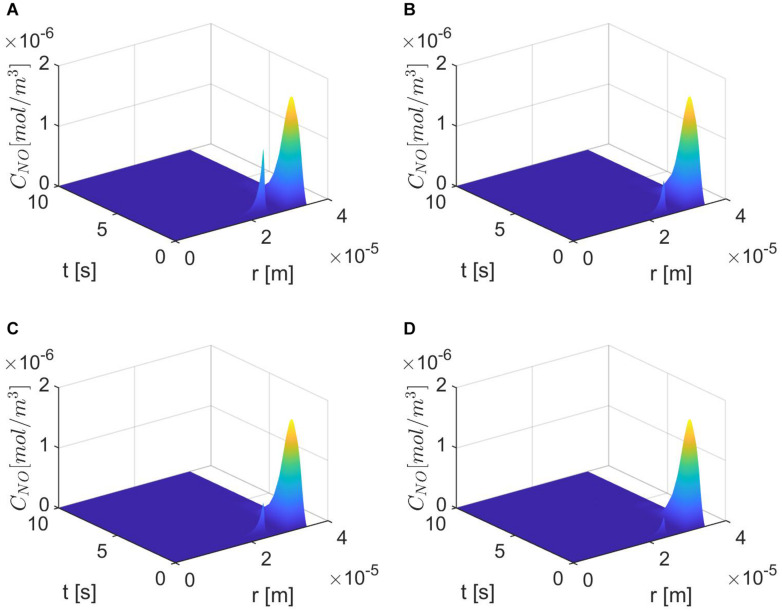
Spatiotemporal variations of *C*_*NO*_ for ε = 0.85, *p* = 0.75 (*q* = 0.25), **(A)** $E=6\times 10^{3}N/m,\gamma =0.75,\tilde{\lambda }=6.5325\times 10^{3}s^{-1}$, **(B)** $E=6\times 10^{4}N/m,\gamma =1.25,\tilde{\lambda }=6.5325\times 10^{3}s^{-1},$ **(C)** *E* = 6×10^3^ *N*/*m*, γ=0.75, $\tilde{\lambda }=13.065\times 10^{3}s^{-1}$, and **(D)** *E* = 6×10^4^ *N*/*m*, γ = 1.25, $\tilde{\lambda }=13.065\times 10^{3}s^{-1}$.

**FIGURE 8:**
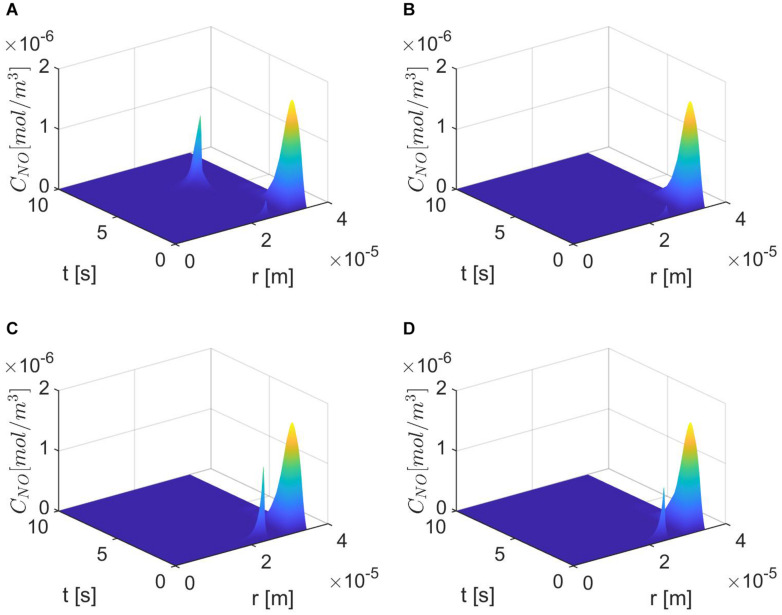
Spatiotemporal variations of *C*_*NO*_ for ε = 0.85, *p* = 0.25 (*q* = 0.75), **(A)** $E=6\times 10^{3}N/m,\gamma =0.75,\tilde{\lambda }=6.5325\times 10^{3}s^{-1}$, **(B)** $E=6\times 10^{4}N/m,\gamma =1.25,\tilde{\lambda }=6.5325\times 10^{3}s^{-1},$ **(C)** *E* = 6×10^3^ *N*/*m*, γ=0.75, $\tilde{\lambda }=13.065\times 10^{3}s^{-1}$, and **(D)** *E* = 6×10^4^ *N*/*m*, γ = 1.25, $\tilde{\lambda }=13.065\times 10^{3}s^{-1}$.

Figures 6–8 show the results for ε = 0.85, two values for *p* (0.75 and 0.25), and various values for *E*, γ, and $\tilde{\lambda }$. In this case, the spatiotemporal variations of the NO concentration show again the highest production of NO happening at the neuronal site (Figures 7, 8). By comparison, the peaks of the NO concentration at the endothelium happen within the same time interval but have narrower shapes. In the case *p* = 0.25, *q* = 0.75, and values for *E*, γ, and $\tilde{\lambda }$ corresponding to normal conditions, the NO concentration at the endothelium has two peaks, with the highest happening later than the peak in the NO concentration at the neuronal site (Figure 8A). For larger values of *E*, γ (stiffer vascular wall), the maximum values of the NO concentration for the two cases *p* = 0.25, *q* = 0.75 and *p* = 0.75, *q* = 0.25 decrease (Figures 6–8). Also, in this scenario, the maximum value of the NO concentration for *p* = 0.25, *q* = 0.75 is lower than the one for *p* = 0.75, *q* = 0.25 (Figures 6, 7B, 8B). Figures 6, 7C, 8C show also lower maximum values of the NO concentration at the endothelium for a larger value of $\tilde{\lambda }$ (bigger amount of superoxide). However, in this scenario, the maximum value of the NO concentration, for *p* = 0.25, *q* = 0.75, is higher than the one for *p* = 0.75, *q* = 0.25 (Figure 6). In the presence of stiffer vascular wall and larger amount of superoxide, the model predicts a decrease in the NO concentration at the endothelium in both cases (*p* = 0.25, *q* = 0.75 and *p* = 0.75, *q* = 0.25) (Figures 6, 7D, 8D). Interestingly, the delayed, second higher peak in the NO concentration at the endothelium happening in the case *p* = 0.25, *q* = 0.75 disappears for larger values of *E*, γ, and/or $\tilde{\lambda }$. Thus, all the peaks of the NO concentration at the endothelium happen fast within the first 2 s, and only in the case when the diffusion of NO in the backward direction is stronger (the case *p* = 0.25, *q* = 0.75) and the values of *E*, γ, and $\tilde{\lambda }$ correspond to normal conditions will a delayed second and higher peak be observed. Last, by comparing Figures 6A,B, it appears that the case *p* = 0.75, *q* = 0.25 predicts the lowest NO concentration when both the amount of superoxide and the wall’s stiffness are higher.

**FIGURE 9:**
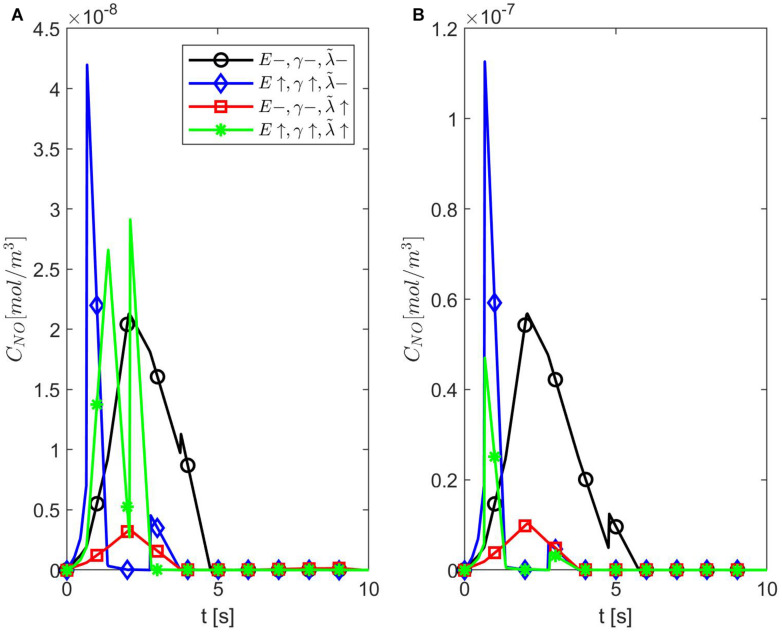
Temporal profiles of *C*_*NO*_ at *r* = *a* + *h*, the outer boundary of the endothelium, for ε = 0.95, **(A)** *p* = 0.75 (*q* = 0.25), and **(B)** *p* = 0.25 (*q* = 0.75) (**A,B** have the same legend shown in **A**). Here $E=6\times 10^{3}N/m,\gamma =0.75,\tilde{\lambda }=6.5325\times 10^{3}s^{-1}$(black circle symbol), $E=6\times 10^{4}N/m,\gamma =1.25,\tilde{\lambda }=6.5325\times 10^{3}s^{-1}$ (blue diamond symbol), $E=6\times 10^{3}N/m,\gamma =0.75,\tilde{\lambda }=13.065\times 10^{3}s^{-1}$ (red square symbol), and $E=6\times 10^{4}N/m,\gamma =1.25,\tilde{\lambda }=13.0655\times 10^{3}s^{-1}$ (green star symbol).

**FIGURE 10:**
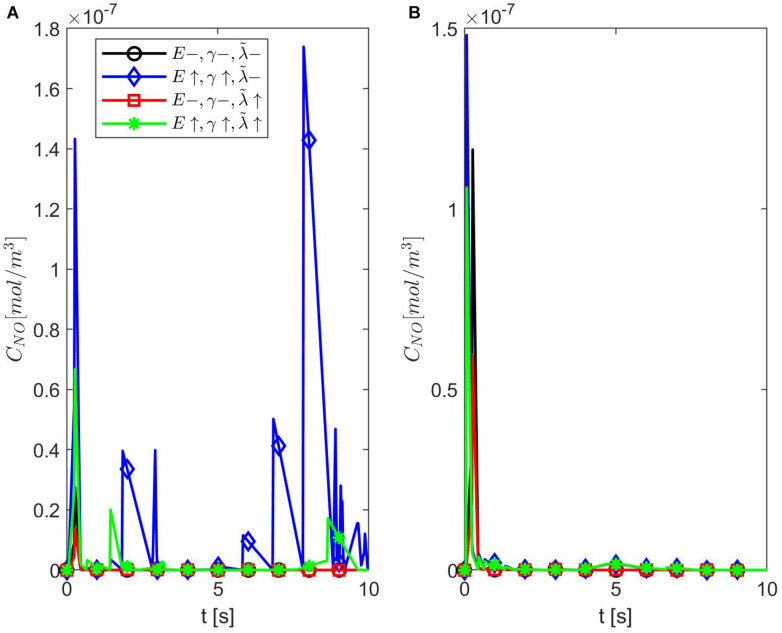
Temporal profiles of *C*_*NO*_ at *r* = *a* + *h*, the outer boundary of the endothelium, for ε = 0.75, **(A)** *p* = 0.75 (*q* = 0.25), and **(B)** *p* = 0.25 (*q* = 0.75) (**A,B** have the same legend shown in **A**). Here $E=6\times 10^{3}N/m,\gamma =0.75,\tilde{\lambda }=6.5325\times 10^{3}s^{-1}$ (black circle symbol), $E=6\times 10^{4}N/m,\gamma =1.25,\tilde{\lambda }=6.5325\times 10^{3}s^{-1}$ (blue diamond symbol), $E=6\times 10^{3}N/m,\gamma =0.75,\tilde{\lambda }=13.065\times 10^{3}s^{-1}$ (red square symbol), and $E=6\times 10^{4}N/m,\gamma =1.25,\tilde{\lambda }=13.0655\times 10^{3}s^{-1}$ (green star symbol).

Figures 9, 10 show the temporal profiles of the NO concentration at the endothelium for ε = 0.95 and, respectively, ε = 0.75 in the same scenarios as those shown above for ε = 0.85. Aside from the expected distribution skewness, the behavior of the NO concentration resembles the one for ε = 1: an increase in the NO concentration with increasing vascular stiffness and a decrease in the NO concentration with a larger quantity of superoxide and/or in the presence of stiffer vascular walls. This suggests that there exists a narrow range of values for ε near 0.85 where the combined effects of anomalous diffusion, stiffer vascular wall, and increased quantity of superoxide contribute to the decrease in the NO concentration at the endothelium reported, for instance, in Chironi et al. (2009). Thus, there exists a characteristic jump length distribution for ε = 0.85 when the expected behavior of the NO concentration at the endothelium is observed in the forward diffusional direction. Last, it is worth mentioning that in all studied cases of NO anomalous diffusion (0.75 ≤ ε < 1) the maximum values of the NO concentration at the vascular wall are within the range of physiological values (Hall and Garthwaite, 2006).

## Discussion

The main predictions of the proposed model of NO dynamics are that, when the fractional order of the NO anomalous diffusion is ε = 0.85 and the forward diffusional direction dominates, the NO concentration at the vascular wall: (1) is maximum when the stiffness of the wall and the amount of superoxide are within normal ranges (Figure 5) and (2) decreases the most in the presence of a stiffer vascular wall and an increased concentration of superoxide (Figure 6A). According to Yan et al. (2003), Densmore et al. (2006), Chironi et al. (2009), Burger et al. (2013), Foley and Conway (2016), and Leopold (2020), the combination of factors mentioned in item 2 above may be caused by an increased concentration of Ang II, while the subsequent reduced amount of endothelial NO contributes to endothelial dysfunction and endotheliopathy. Although at this stage the model is rather general and does not include specifics of a particular disease, it could potentially be adapted to studies of any disease that causes endotheliopathy according to the mechanism described above. Thus, for now, only a big picture of the usefulness of the proposed model will be provided. The discussion that follows will focus on COVID-19 because of the fast evolution of the disease (compared with other cardiovascular and neurodegenerative diseases, which can take years to develop symptoms and be diagnosed) and the ongoing high interest in its treatment.

As mentioned in the section “Introduction” (see also Figure 1), a higher amount of Ang II is linked to the infection with the SARS-CoV-2 virus. The relationship among the SARS-CoV-2 virus infection, endotheliopathy, and NO was investigated in Akaberi et al. (2020), Bagate et al. (2020) and Goshua et al. (2020). For instance, the blood test results of patients with COVID-19 that are presented in Goshua et al. (2020) provide a biochemical proof of endotheliopathy presence. The analysis of several endothelial markers in the blood highlights the important role played by endotheliopathy in disease severity and the survival of patients with COVID-19. Endothelial cell modification was, thus, suggested as one additional therapy that may improve the outcome of some critically ill patients. The studies in Akaberi et al. (2020) and Bagate et al. (2020) looked at NO delivery as a potential therapy for COVID-19. The *in vitro* experiments reported in Akaberi et al. (2020) were performed on cultured cells, which were infected with the SARS-CoV-2 virus and then exposed to two pharmacological NO-evolving agents (SNAP and NAP). The results showed that the NO donor SNAP inhibited the replication of the virus. On the other hand, the small clinical study in Bagate et al. (2020) shows that, although the combination of inhaled NO and almitrine, a pulmonary vasoconstrictor, rapidly improved the arterial oxygenation in intubated patients with COVID-19, the benefits of this therapeutical approach were temporary and did not have a positive impact on outcome. Nevertheless, this method of NO administration could be used in some patients with COVID-19 in need of urgent care, while other NO-evolving pharmaceutical agents could be added to the treatment of COVID-19 and possibly become part of a long-term plan of supporting the endothelial function after recovery from COVID-19. More details on various pharmaceutical agents modulating the NO bioavailability that could benefit patients with COVID-19 can be found in Ignarro et al. (2002) and Kraehling and Sessa (2017).

Two challenges that are encountered during the design of a treatment are the diligent selection of patients who will benefit from the treatment and the careful planning of therapeutical delivery that will improve patient outcome. The model proposed in this article might provide some guidance on how to approach these therapeutical challenges for COVID-19. Numerical simulations suggest the existence of a critical state when a stiffer vessel’s wall, increased amount of superoxide, and specific value of the NO anomalous diffusion exponent ε leads to a very low concentration of endothelial NO in the brain. This low concentration might be used as a threshold value in images of NO concentration inside and outside the endothelial cells (as well as other brain cells). For instance, certain fluorescein biosensors can be used as NO probes for real-time visualization of the spatiotemporal distribution of NO in fluoroscopy (Sharma et al., 2014), while manganese-based NO-selective contrast agents can help with magnetic resonance images (MRI) of NO (Barandov et al., 2020). These biosensors are chemical agents that could be delivered to the brain either via the intracerebrospinal fluid or by intravascular injection. Also, methods that reduce the neurotoxicity of these NO probes, so that they are safe to use in human subjects, are emerging (Sudarshana et al., 2019). However, an image showing a low NO concentration alone or in combination with a measurement of stiffer retinal vessels done during an eye exam (Best et al., 1971; Flammer et al., 2013) will just indicate the presence of endotheliopathy in patients with COVID-19, which, as mentioned earlier, can already be found with blood tests.

Imaging anomalous diffusion of NO could help not only with the selection of patients who will benefit from the administration of NO-evolving pharmaceutical agents but also with the design of optimal drug delivery. For instance, the diffusion-weighted magnetic resonance spectroscopy (DW-MRS) method proposed in Marchadour et al. (2012) could be adapted to NO by changing the spectroscopy and diffusion sequences such that the pseudo diffusion coefficient *D*^∗^ given in Zhou et al. (2010) [see also Magin et al. (2008) for more details) to model the measured apparent diffusion coefficient (ADC) may be used:

(13) $$D^{*}=D^{\frac{1}{\beta }}\mu^{\frac{2\left(\beta -1\right)}{\beta }}{\left(\Delta -\frac{2\beta -1}{2\beta +1}\delta \right)}^{\frac{1-\beta }{\beta }}$$

where, δ and Δ are the diffusion gradient pulse width and gradient lobe separation, respectively. Parameters *D*, μ, and β are introduced in the space-fractional Bloch–Torrey equation of order 2β ∈ (0, 2] for the transverse magnetization and can be found by fitting formula (13) to the measured ADC. Formula (13) was used in Zhou et al. (2010) to show anomalous diffusion in human brain using DW-MRI. If a relationship between the fractional orders β and ε may be established, then the value of β corresponding to ε = 0.85 could be used to select patients with COVID-19 for treatment with NO-evolving pharmaceutical agents. Also, the model predicts that, for ε = 0.85, the NO concentration has a maximum value at the vascular wall when the vascular stiffness and amount of superoxide are within normal ranges, and thus, the NO signal is better in this case. This suggests that a younger, otherwise healthy, subject diagnosed with COVID-19 soon after the onset of symptoms could benefit from a DW-MRS of NO, ideally done shortly after diagnosis, since a value of β corresponding to ε = 0.85 may indicate that the subject is at a higher risk of developing endotheliopathy. Furthermore, this critical value of β may be useful in accurately programming drug delivery so that maximum therapeutical benefits are achieved. For instance, in Ojha et al. (2020), it is shown how some biocompatible materials can be engineered such that immersed nanoparticles follow specific anomalous diffusion patterns. This approach can be used to control the release of pharmaceutical agents by nanoparticle drug carriers. Thus, it is possible that, for example, the optimal delivery of NO-evolving drugs happens when a certain relationship between the anomalous diffusion exponent of the nanoparticle carrier and β (or ε) holds. Last, it is important to mention that the proposed methodology for COVID-19 is purely exploratory since the required technology and critical knowledge about this novel disease are still missing.

The main limitations of the proposed model are as follows. The values of most parameters given in Table 1 were taken from published theoretical studies (Vaughn et al., 1998a; Kavdia et al., 2002; Hall and Garthwaite, 2006; Hodis and Zamir, 2008), which are still to be validated through *in vivo* experiments. Some other important biochemical processes that are involved in the production and decay of cerebral NO [see for instance, Attwell et al. (2010)], such as *O*_2_− modulated synthesis of NO and NO inactivation due to autoxidation, were not incorporated into the model. The mechanical deformation of the vascular wall was studied in a simplified, one-dimensional geometry, and the blood flow was represented as a mere oscillatory boundary condition. None of the mechanical parameters of the vascular wall was assumed to be dependent on the NO concentration. Last, the proposed model of cerebral NO biotransport was one dimensional.

## Conclusion and Future Work

In this article, a space-fractional reaction–diffusion equation for the NO concentration was proposed to model the biotransport of NO in the brain. The model incorporates the NO production by synthesis in the neurons and by shear-induced mechanotransduction at the endothelium–lumen interface. The neuronal NO synthesis is assumed to have the time-dependent profile of the Ca^2+^-calmodulin binding (Hall and Garthwaite, 2006), while the endothelial NO production is proportional to the viscous dissipation in the endothelium. The endothelium is assumed to be a homogeneous, isotropic Maxwell linear viscoelastic material exposed to the pulsatile blood flow (Hodis and Zamir, 2008). The NO inactivation is caused, among others, by the blood’s hemoglobin in the lumen and by the reaction with superoxide in the extracellular space. The NO diffusion is assumed to be anomalous due to the trapping of the NO particles by the EMP whose concentration increases in pathological conditions. Thus, the presence of EMP is modeled as anomalous diffusion of NO. The left- and right-sided Riemann–Liouville fractional derivatives of order ε ∈ (0, 1) are used to model anomalous diffusion. Numerical simulations show that there exists a characteristic jump length distribution for ε = 0.85 where the NO concentration at the vascular wall decreases in the presence of a stiffer vascular wall and larger amount of superoxide. The biggest distribution skewness of the NO at the wall was also noticed for the same value of ε.

Future work will focus on resolving the limitations of the proposed model mentioned in the previous section. A full sensitivity analysis to parameters’ changes will be performed, and other components shown in Figure 1 as well as biochemical processes specific to the infection with the SARS-CoV-2 virus will be incorporated into the model. As more clinical and experimental data on COVID-19 are becoming available in the literature, the validation of an improved, disease-specific model should, thus, be possible. Last, the model will be generalized to a three-dimensional geometry and will couple the blood flow, the deformations of the vessel’s wall, and the NO dynamics. The mechanical parameters introduced by the constitutive equations of the blood and the wall will be assumed to depend on the NO concentration. In addition, the intriguing idea proposed in Helms and Kim-Shapiro (2013) that the hemoglobin plays a dual role of destroyer and preserver of the NO bioactivity will be explored through updated mathematical representations of the NO production and decay in the three-dimensional expression of equation (1). Such a mathematical model could not only provide a better understanding of the impacts of COVID-19 on the cardiovascular and nervous systems but also contribute to the development of successful NO-based therapies for brain diseases associated with disturbances in NO biotransport and/or signaling.

## Data Availability Statement

The original contributions presented in the study are included in the article/supplementary material, further inquiries can be directed to the corresponding author.

## Author Contributions

AT wrote the MATLAB code, generated the numerical simulations, and contributed to the interpreting the results and writing of the article. CD developed the mathematical model, proposed the linkage between SARS-Cov-2 and NO, and contributed to the interpreting of the results and writing of the article. Both authors contributed to the article and approved the submitted version.

## Conflict of Interest

The authors declare that the research was conducted in the absence of any commercial or financial relationships that could be construed as a potential conflict of interest.
